# Functionalization of heterocyclic compounds using polyfunctional magnesium and zinc reagents

**DOI:** 10.3762/bjoc.7.147

**Published:** 2011-09-13

**Authors:** Paul Knochel, Matthias A Schade, Sebastian Bernhardt, Georg Manolikakes, Albrecht Metzger, Fabian M Piller, Christoph J Rohbogner, Marc Mosrin

**Affiliations:** 1 Department Chemie, Ludwig-Maximilians-Universität München, Butenandtstr. 5-13, 81377 München, Germany

**Keywords:** cross-coupling, heterocycles, insertion, metalation, organomagnesium, organozinc

## Abstract

In this review we summarize the most important procedures for the preparation of functionalized organzinc and organomagnesium reagents. In addition, new methods for the preparation of polyfunctional aryl- and heteroaryl zinc- and magnesium compounds, as well as new Pd-catalyzed cross-coupling reactions, are reported herein. Experimental details are given for the most important reactions in the Supporting Information File 1 of this article.

## Introduction

The functionalization of heterocyclic scaffolds is an important task in current pharmaceutical research. In this review article, we describe the approaches to this problem that use functionalized magnesium and zinc heterocyclic intermediates. Some typical experimental procedures are indicated in each case for the most important methods. New Pd-catalyzed cross-coupling procedures are also presented.

## Review

### 1 Preparation of heterocyclic zinc reagents

Organozinc compounds [1–3] are important synthetic intermediates as they are compatible with a broad range of functional groups. The reactivity of a carbon–zinc bond is quite low, and therefore, reactions with organic electrophiles often require the use of transition metal catalysts. The preparation of aryl and heteroaryl zinc derivatives is conveniently achieved by three general procedures:

- the direct insertion of zinc dust to aryl or heteroaryl iodides or bromides;
- the direct insertion of magnesium in the presence of Zn(II) salts to aryl or heteroaryl halides;
- the metalation of aryl or heteroaryl derivatives with TMP_2_Zn·2MgCl_2_·2LiCl.

**Scheme 1:**
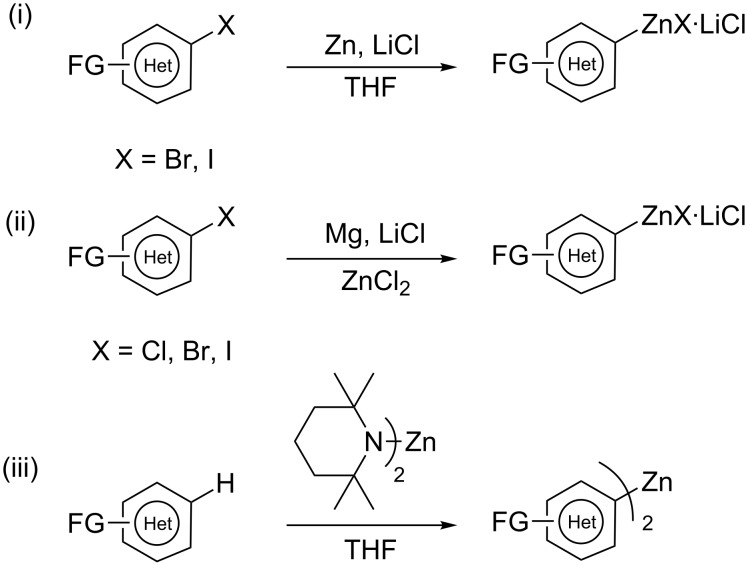
Preparation of polyfunctional heteroarylzinc reagents.

These three methods, developed recently in our laboratories, provide access to numerous heterocyclic zinc reagents (Scheme 1).

#### 1.1 The direct insertion of zinc in the presence of LiCl

**Scheme 2:**
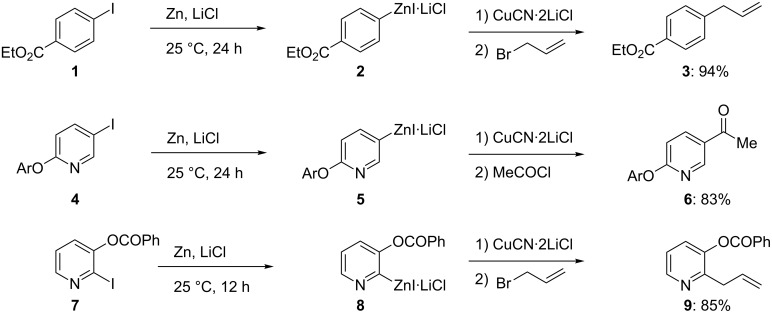
LiCl-mediated insertion of zinc dust to aryl and heteroaryl iodides.

Although the direct insertion of zinc dust to alkyl iodides proceeds readily, the insertion to aryl iodides is very slow in THF and requires the use of polar solvents [4] or highly activated zinc [5]. Recently, we found that the presence of LiCl greatly facilitates the insertion of zinc to aryl iodides [6]. Thus, the insertion of zinc dust (activated with 1,2-dibromoethane and Me_3_SiCl) to ethyl 4-iodobenzoate (**1**) at 70 °C provides less than 5% of zinc reagent **2** after a reaction time of 24 h. On the other hand, in the presence of one equivalent of LiCl, the insertion of zinc is completed within 24 h at 25 °C. After the addition of a catalytic amount of CuCN·2LiCl [7], the arylzinc intermediate is allylated with allyl bromide providing the ester **3** in 94% isolated yield (Scheme 2) [6].

**Scheme 3:**
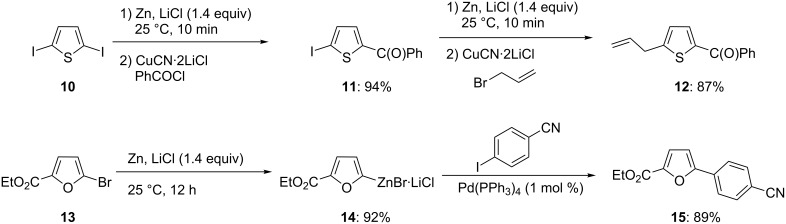
Selective insertions of Zn in the presence of LiCl.

This method can be extended to a broad variety of functionalized heterocyclic iodides such as the pyridines **4** and **7**. The corresponding zinc reagents **5** and **8** are obtained at 25 °C in quantitative yield. The allylation of pyridylzinc derivative **8** with allyl bromide provides pyridine **9** in 85% yield [6]. Interestingly, a diiodide, such as 2,5-diiodothiophene (**10**) reacts selectively with Zn and LiCl to provide the iodothienyl ketone **11** in 94% yield after benzoylation. Subsequent treatment of **11** with a second amount of Zn and LiCl (1.4 equiv) provides a new intermediate zinc reagent within 10 min, which after allylation provides the 2,5-disubstituted thiophene **12** in 87% yield (Scheme 3) [6]. The insertion reaction proceeds best with aryl and heteroaryl iodides, however, the presence of electron-withdrawing substituents greatly accelerates the zinc insertion rate and electron-poor-heteroaryl bromides, such as the bromofuran **13**, react smoothly with Zn and LiCl to furnish the furylzinc reagent **14** within 12 h at 25 °C, which after Pd-catalyzed cross-coupling (Negishi reaction) affords the 5-arylated furan **15** in 89% yield.

**Scheme 4:**
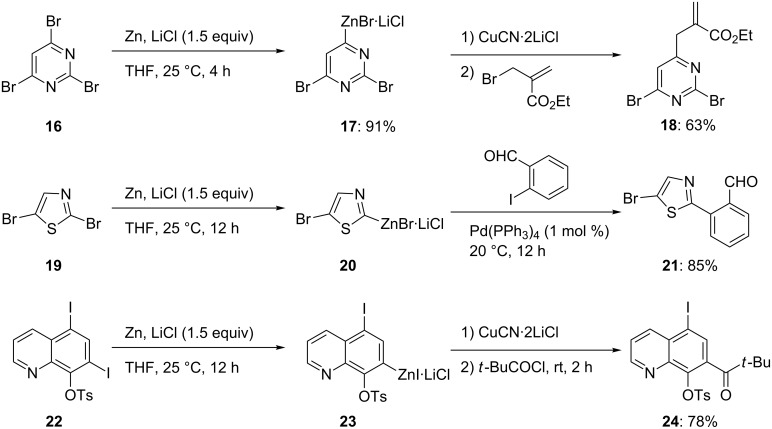
Chemoselective insertion of zinc in the presence of LiCl.

Interestingly, a high chemoselectivity is observed with several heterocyclic dihalides [8–9]. Thus, the tribromopyrimidine **16** provides only the 4-zincated pyrimidine **17**. After allylation, the expected allylated pyrimidine **18** is obtained in 63% yield. Also, the dibromothiazole **19** allows insertion of zinc only into the most labile C–Br bond (in position 2) leading to the zincated thiazole **20**. After Negishi cross-coupling [10–12], the 2-arylated thiazole **21** is obtained in 85% yield. Polar functional groups, such as a tosyloxy-group are able to direct the zincation. Thus, the diiodoquinoline **22** is regioselectively zincated (25 °C, 12 h) to intermediate **23** leading to the polyfunctional quinoline **24** in 78% yield after copper(I)-mediated acylation (Scheme 4 and Supporting Information File 1, Procedure 1) [8]. This regioselectivity is explained by the polar and electron-poor nature of the tosyloxy group, which leads to a strong electron-withdrawing effect and accelerates the insertion of zinc into the neighboring C–I bond. The presence of LiCl amplifies this effect through coordination to the tosyloxy group and to the *ortho*-iodide, and therefore facilitates the cleavage of this carbon–iodide bond.

**Scheme 5:**
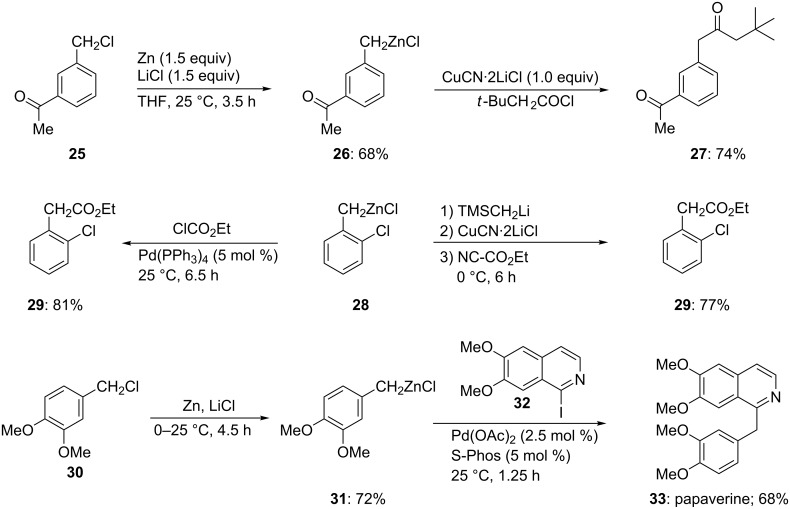
Preparation and reactions of benzylic zinc reagents.

This method has been extended to the preparation of benzylic zinc reagents [13]. A remarkable chemoselectivity is observed and functional groups, such as an acetyl group, are perfectly compatible with such synthesis. Thus, the reaction of the benzylic chloride **25** with zinc dust (1.5 equiv) and LiCl (1.5 equiv) in THF at 25 °C for 3.5 h provides the corresponding zinc reagent **26** in 68% yield. Its half-life at 25 °C is approximately two days. The copper(I)-mediated acylation of **26** provides the expected diketone **27** in 74% yield (Scheme 5) [13–14].

**Scheme 6:**
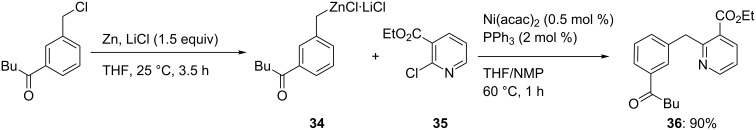
Ni-catalyzed cross-coupling of benzylic zinc reagent **34** with ethyl 2-chloronicotinate.

A broad range of functional groups are tolerated, and homocoupling products account for less than 5% of the total. These benzylic zinc reagents give access to biologically important phenylacetic acids. Thus, the treatment of the chloro-substituted benzylic zinc compound **28** with ClCO_2_Et in the presence of Pd(PPh_3_)_4_ (5 mol %) furnishes the phenylacetic derivative **29** in 81% yield. Alternatively, a copper (I)-mediated reaction with NC-CO_2_Et provides the same product in 77% yield when a dummy ligand is added (Scheme 5) [13]. Electron-rich benzylic chlorides, such as **30** are also readily converted to the desired zinc reagents **31**. The Pd-catalyzed cross-coupling of **31** with the iodoquinoline **32** and with S-Phos as ligand [15–17] provides the alkaloid papaverine (**33**) in 68% yield (Scheme 5) [13]. Ni-catalyzed cross-couplings can also be realized [14]. Thus, the reaction of the benzylic zinc reagent **34**, obtained by direct zinc insertion in the presence of LiCl, with the chloropyridine **35** in the presence of Ni(acac)_2_ (0.5 mol %) and PPh_3_ (2 mol %) affords the polyfunctional pyridine **36** in 90% yield (Scheme 6 and Supporting Information File 1, Procedure 2) [14,18–19].

#### 1.2 The direct insertion of magnesium in the presence of ZnCl_2_: A new preparation of unsaturated zinc reagents bearing sensitive functionalities

**Scheme 7:**
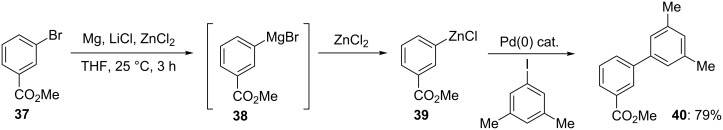
In situ generation of arylzinc reagents using Mg in the presence of LiCl and ZnCl_2_.

Although the LiCl-mediated zinc insertion represents a major preparative advance for the synthesis of polyfunctional zinc reagents, this method has an intrinsic limitation due to the use of zinc as a reducing agent. Zinc has only moderate reducing properties, therefore its insertion into organic halides only proceeds smoothly in the case of aryl iodides and electron-poor substituted aryl bromides. The use of highly reactive zinc (Rieke-zinc) [20–21] solves the problem only partially. It is expensive and most aryl or heteroaryl chlorides do not react. Therefore, we used a stronger reducing reagent, magnesium. Magnesium turnings readily insert into a variety of aryl chlorides or bromides in the presence of LiCl. However, arylmagnesium reagents are compatible with fewer functional groups. Thus, methyl esters react rapidly with arylmagnesium reagents at 0 °C. In order to solve this problem, we have performed a Barbier-type preparation of aryl and heteroaryl zinc reagents by treating the aryl bromide or chloride with magnesium turnings in the presence of zinc chloride and LiCl. Under these conditions, the relatively unreactive aryl bromides and chlorides readily react. Furthermore, sensitive functionalities are tolerated since the reactive arylmagnesium species generated is immediately trapped with zinc chloride (Scheme 7) [22]. Thus, methyl 3-bromobenzoate (**37**) reacts with magnesium powder in the presence of LiCl (1.5 equiv) and ZnCl_2_ (1.1 equiv) to provide the intermediate magnesium species **38**, which is immediately trapped with ZnCl_2_ leading to the zinc reagent **39** in high yields. Subsequent Pd-catalyzed cross-coupling of **39** with an aryl iodide provides the cross-coupling product **40** in 79% yield (Scheme 7) [22].

#### 1.3 Preparation of heteroaryl zinc reagents by direct zincation of heterocyclic compounds using the new zinc base TMP_2_Zn·2MgCl_2_·2LiCl (**42**)

**Scheme 8:**
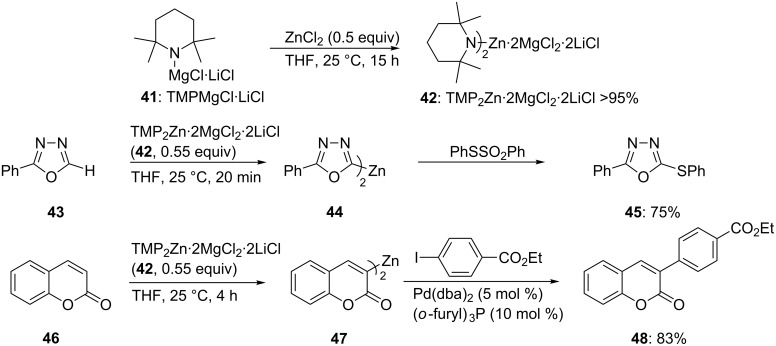
Zincation of heterocycles with TMP_2_Zn (**42**).

The preparation of zinc reagents by a directed deprotonation was of limited use as no soluble zinc base was available [23–24]. We found that the treatment of commercially available TMPMgCl·LiCl (**41**) [25–27] with ZnCl_2_ (0.5 equiv) at 25 °C provides the new base TMP_2_Zn·2MgCl_2_·2LiCl (**42**) [28]. All three metals Zn, Mg and Li are important in this mixed base [29]. The role of LiCl is to increase the solubility of the base, the role of MgCl_2_ is to increase its reactivity and the role of zinc is essential since it confers to this base an exceptional chemoselectivity (Scheme 8). Thus, the 1,3,4-oxadiazole **43** is readily converted to the zinc reagent **44** by the reaction with TMP_2_Zn·2MgCl_2_·2LiCl (**42**, 0.55 equiv; 25 °C, 20 min). It should be noted that both TMP-moieties are used and that no fragmentation of this sensitive heterocycle is observed, as is the case for the corresponding Mg- and Li-derivatives.

**Scheme 9:**
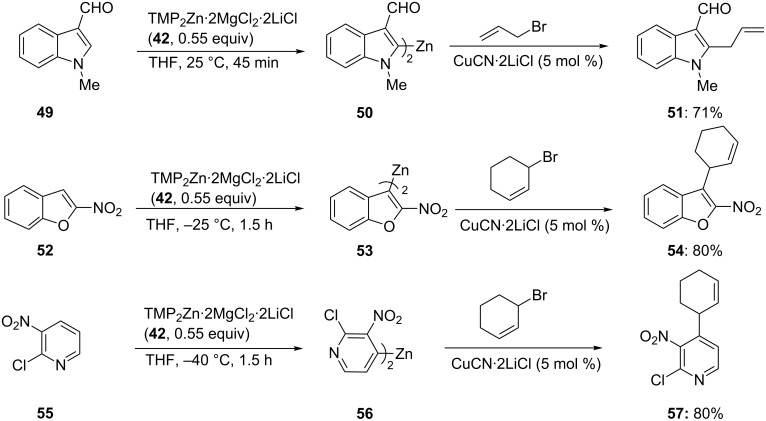
Preparation of highly functionalized zincated heterocycles using TMP_2_Zn·2MgCl_2_·2LiCl (**42**).

After a reaction of the heterocyclic zinc reagent **44** with PhSO_2_SPh the corresponding thio-derivative **45** is obtained in 75% yield. Coumarine (**46**) can be directed zincated leading to the zinc reagent **47**. After a Negishi cross-coupling with an aromatic iodide, the substituted coumarine **48** is obtained in 83% yield (Scheme 8 and Supporting Information File 1, Procedure 3) [28]. This procedure tolerates most of the important functional groups in organic chemistry. Thus, the zincation of the formyl-substituted indole **49** is complete within 45 min at 25 °C leading to the zinc reagent **50**. After allylation, the 2,3-disubstituted indole **51** is obtained in 71% yield (Scheme 9). Similarly, 2-nitrobenzofuran (**52**) is zincated without reacting with the nitro group, leading to the nitro-substituted zinc reagent **53**. After allylation, the benzofuran **54** is obtained in 80% yield. The polyfunctional pyridine **55** is zincated with TMP_2_Zn·2MgCl_2_·2LiCl (**42**) leading to the zinc reagent **56**. Subsequent allylation furnishes the trisubstituted pyridine **57** in 80% yield (Scheme 9) [28].

**Scheme 10:**
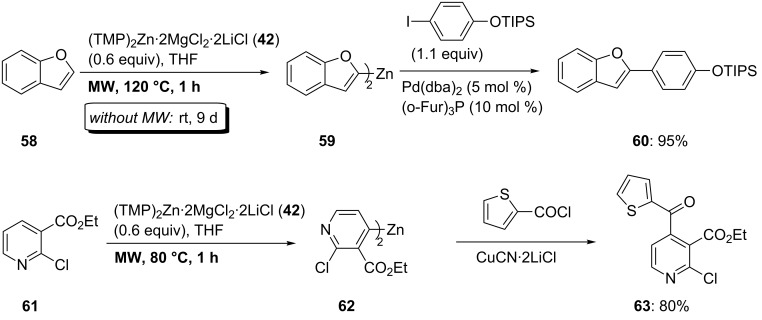
Microwave-accelerated zincation of heterocycles using TMP_2_Zn·2MgCl_2_·2LiCl (**42**).

In some cases, the zincation using TMP_2_Zn·2MgCl_2_·2LiCl (**42**) is slow and requires long reaction times. This is the case for benzofuran (**58**), which requires 9 days at 25 °C for a complete zincation in position 2 leading to **59**. The reaction time can be dramatically decreased by means of microwave irradiation. Under these conditions, the zincation is complete within 1 h at 120 °C (Scheme 10). Similarly, the functionalized pyridine **61** is zincated within 1 h at 80 °C under microwave irradiation leading to **62**. The success of this procedure is a result of the high thermal stability of organozinc reagents. A Pd-catalyzed cross-coupling of **59** or a copper(I)-mediated acylation of **62** affords the products **60** and **63** in 80–95% yield (Scheme 10 and Supporting Information File 1, Procedure 4) [30].

### 2 Preparation of heterocyclic magnesium reagents

Unexpectedly, recent research work from our laboratories showed that the preparation of heteroarylmagnesium reagents is compatible with numerous functional groups [31–33]. There are three important synthetic methods for the preparation of polyfunctional heteroarylmagnesium reagents:

1. the bromine- (or iodine-) magnesium exchange reaction;
2. the direct insertion of magnesium turnings in the presence of LiCl;
3. the direct magneziation of heterocycles using TMPMgCl·LiCl (**41**).

Due to the higher polarity of the carbon–magnesium bond, these heterocyclic organometallics are significantly more reactive than the corresponding zinc reagents. This makes their preparation especially important.

#### 2.1 The preparation of heterocyclic magnesium reagents through a bromine- (or iodine-) magnesium exchange

**Scheme 11:**
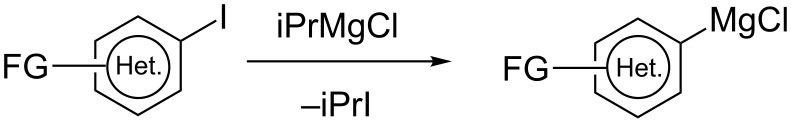
The I/Mg-exchange as a metal-metathesis reaction.

Compared to the halogen/lithium exchange, discovered in 1939 by Wittig and Gilman, the halogen/magnesium exchange is much slower. Whereas aryl and electron-poor unsaturated iodides readily react with iPrMgCl and undergo a metal-metathesis to provide the more stable heteroarylmagnesium reagent (Scheme 11) [34], the reaction of aryl and heteroaryl bromides is slow when iPrMgCl is used as an exchange reagent.

**Scheme 12:**
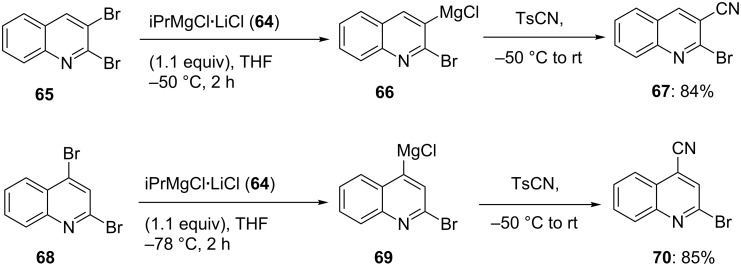
Regioselective Br/Mg-exchange of dibromoquinolines **65** and **68**.

However, with the aid of the mixed Li/Mg-reagent iPrMgCl·LiCl (**64**), an efficient exchange reaction is also effective with a wide range of aryl and heteroaryl bromides [31–33,35]. This reagent (**64**) is commercially available as an approx. 1 M THF solution from Chemetall GmbH [27]. Recently, we have applied this exchange reaction for the regioselective functionalization of quinolines. Thus, the 2,3-dibromoquinoline (**65**) is regioselectively converted to the 3-magnesiated quinoline derivative **66**. Using the same exchange reagent, iPrMgCl·LiCl (**64**) and 2,4-dibromoquinoline (**68**), it is now possible to obtain the 4-magnesiated quinoline **69**. All these magnesiations proceed at low temperature (−50 °C to −78 °C) and are complete within 2 h reaction time. After reaction with TsCN, the corresponding nitriles **67** and **70** were obtained in 84–85% yield (Scheme 12 and Supporting Information File 1, Procedure 5) [36].

**Scheme 13:**
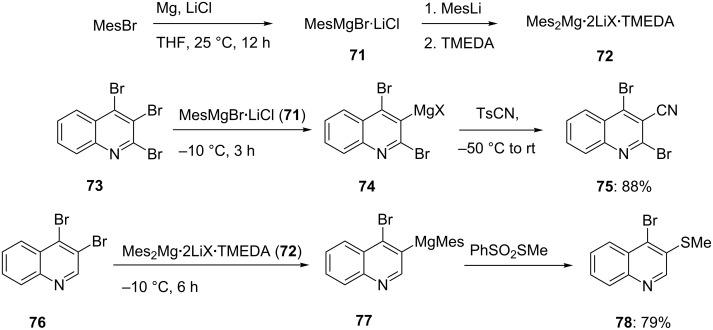
Improved reagents for the regioselective Br/Mg-exchange on bromoquinolines.

The rate of the Br/Mg-exchange depends on the electronic density of the heterocyclic rings. The electron-poor ring systems undergo considerably faster Br/Mg-exchange reactions than do heterocyclic ring systems bearing electron-rich substituents [31–35]. Therefore, in order to achieve a regioselective exchange with the very electron-poor tribromoquinoline **73**, it was necessary to reduce the reactivity of the exchange reagent and thus, to switch from iPrMgCl·LiCl (**64**) to the less reactive mesitylmagnesium reagent MesMgCl·LiCl (**71**). This reagent is readily prepared by the reaction of mesityl bromide with magnesium turnings in the presence of LiCl (25 °C, 12 h; Scheme 13) [36]. The lower reactivity of **71** allows a perfectly regioselective exchange reaction of **73**, to afford the 3-magnesiated quinoline **74** only. A differentiation between the reactivity of a 3-bromo- and a 4-bromo-substituted quinoline is more difficult and even the use of the less reactive exchange reagent MesMgBr·LiCl is not satisfactory. This reactivity can be further tuned: First by preparing the dimesitylmagnesium reagent Mes_2_Mg·2LiBr (which has a higher reactivity than **71**) and then by adding a complexation reagent, such as TMEDA (1 equiv), which considerably lowers the reactivity [37–38]. The new resulting reagent Mes_2_Mg·2LiBr·TMEDA (**72**) now reacts smoothly with 3,4-dibromoquinoline (**76**) providing selectively the 3-magnesiated 4-bromoquinoline **77**. The quenching of **74** and **77** with TsCN and PhSO_2_SMe, respectively, leads to the regioselectively functionalized quinolines **75** and **78** in 79–88% yield (Scheme 13) [36].

This fine tuning is usually not necessary and numerous Br/Mg-exchange reactions making use of the commercially available reagent iPrMgCl·LiCl (**64**) have been reported in the literature [31–34,39].

**Scheme 14:**
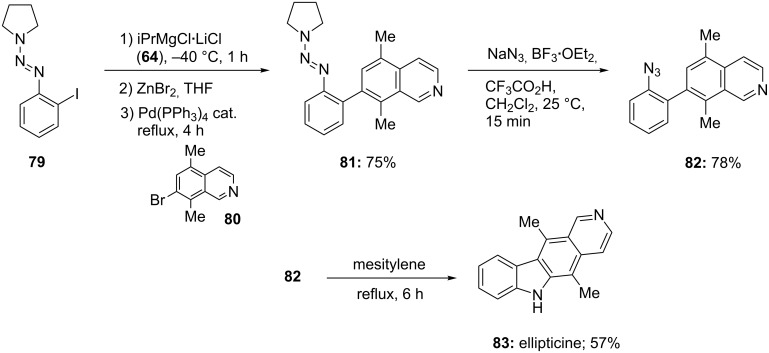
Synthesis of ellipticine (**83**) using an I/Mg-exchange reaction.

The use of iPrMgCl·LiCl also proves to be very practical for the generation of polyfunctional alkenylmagnesium reagents, which react only slowly with iPrMgCl [40–41], as well as for the preparation of arylmagnesium reagents bearing sensitive functionalities, such as triazene. Thus, aryl iodide **79** is treated with iPrMgCl·LiCl (**64**) at −40 °C for 1 h leading to an intermediate magnesium reagent, which after transmetalation to the corresponding zinc reagent using ZnBr_2_ provides, after Negishi cross-coupling reaction with the bromoquinoline **80**, the polyfunctinal triazene **81** in 75% yield. The conversion of the triazene functionality to an azide group is readily achieved by treating **81** with NaN_3_/BF_3_·OEt_2_-CF_3_CO_2_H in CH_2_Cl_2_ leading to the aryl azide **82** in 78% yield. Heating of **82** in mesitylene at reflux for 6 h provides ellipticine **83**, a potent antitumor agent in 57% yield (Scheme 14) [42].

**Scheme 15:**
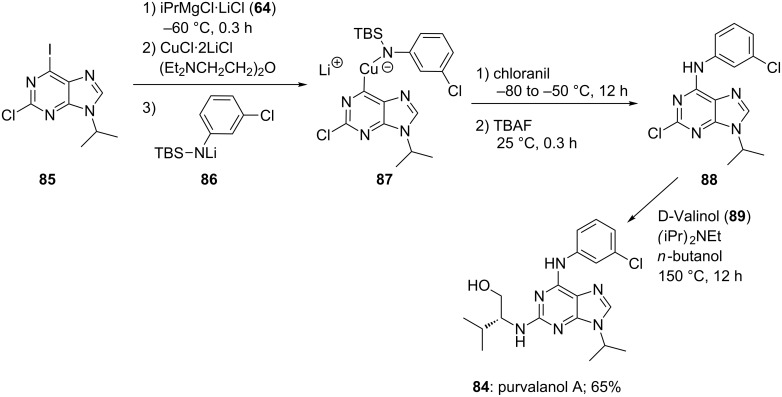
An oxidative amination leading to the biologically active adenine, purvalanol A (**84**).

The structural variations of pyrimidines and purines are very important for the design of antiviral agents. The amination of this class of heterocycles is of particular importance. Recently, we developed an oxidative amination procedure for lithium derivatives using chloranil as oxidation agent [43]. We applied this procedure in the preparation of a CDK inhibitor, purvalanol A (**84**). Thus, an I/Mg-exchange on the purine **85** with iPrMgCl·LiCl (**64**), followed by the transmetalation to the corresponding copper derivative with CuCl·2LiCl, and the addition of the lithiated aniline derivative **86**, furnishes the amidocuprate **87**. In the presence of chloranil amidocuprate **87** undergoes an oxidative coupling providing the adenine derivative **88** in 71% yield. A treatment with D-valinol (**89**) affords the desired CDK inhibitor, purvalanol A (**84**) in 65% yield (Scheme 15) [44].

#### 2.2 The preparation of polyfunctional heterocyclic magnesium reagents by the insertion of Mg in the presence of LiCl

**Scheme 16:**
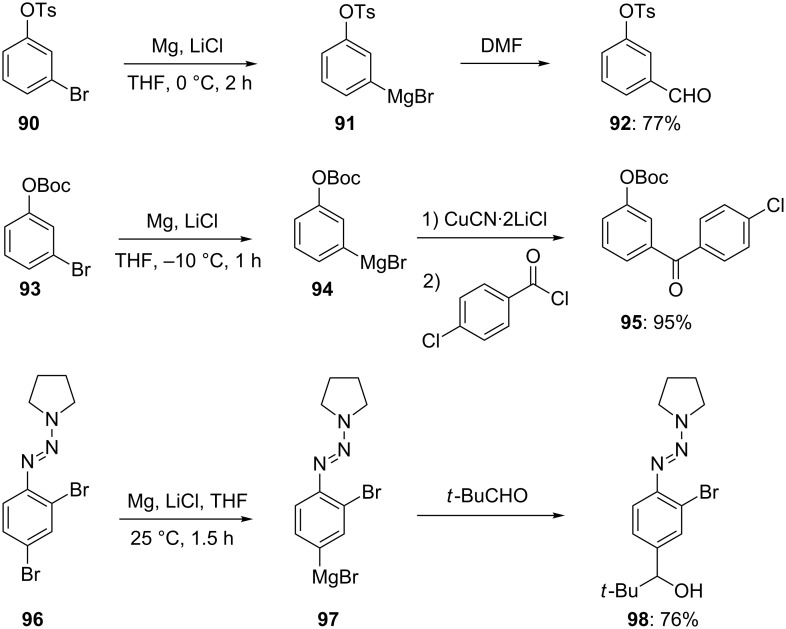
Preparation of polyfunctional arylmagnesium reagents using Mg in the presence of LiCl.

The presence of LiCl facilitates greatly the insertion of many metals into carbon-halogen bonds and avoids the use of expensive activated forms of Mg, such as “Rieke-magnesium”. This property of LiCl for accelerating the insertion of Mg to organic halides has found numerous applications in the preparation of new polyfunctional arylmagnesium reagents. Thus, the rapid reaction of Mg/LiCl with aryl bromides **90**, **93** and **96** allows an expeditive synthesis of the new arylmagnesium derivatives **91**, **94** and **97**. Quenching with typical electrophiles provides the expected products **92**, **95** and **98** in 76–95% yield (Scheme 16) [22,45].

**Scheme 17:**
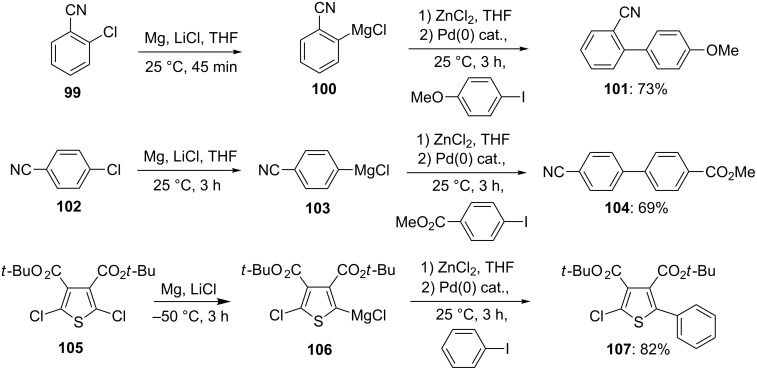
Preparation of polyfunctional magnesium reagents starting from organic chlorides.

Remarkably, this insertion proceeds also with readily available and inexpensive aryl and heteroaryl chlorides, such as **99**, **102** and **105**, providing the functionalized magnesium reagents **100**, **103** and **106** under mild conditions. The cross-coupling reaction of these Grignard reagents and transmetalation to zinc organometallics with ZnCl_2_ affords the expected products **101**, **104** and **107** in 69–82% (Scheme 17 and Supporting Information File 1, Procedure 6) [9,22].

#### 2.3 Preparation of polyfunctional heterocyclic magnesium reagents by directed magnesation using TMPMgCl·LiCl (**41**) or TMP_2_Mg·2LiCl (**129**)

**Scheme 18:**
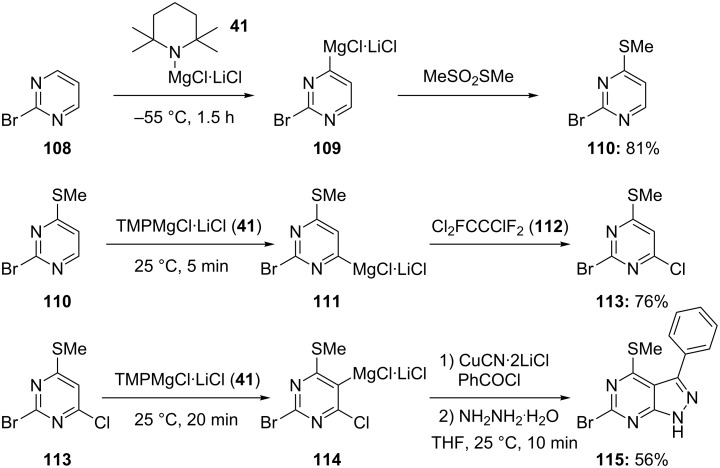
Selective multiple magnesiation of the pyrimidine ring.

The directed magnesiation of aromatic substrates using TMPMgCl·LiCl (**41**) constitutes an economical preparation of a range of functionalized arylmagnesium compounds [25–26]. Sensitive heterocycles such as pyrimidines can be readily magnesiated with commercially available TMPMgCl·LiCl (**41**) [27]. Thus, electron-poor 2-bromopyrimidine (**108**) is converted within 1.5 h at −55 °C in the presence of TMPMgCl·LiCl (**41**) to the corresponding magnesium reagent **109**. A low reaction temperature is required in this case, since the sensitive heterocycle **108** undergoes ring addition reactions at temperatures above −30 °C. Quenching of the 4-magnesiated pyrimidine **109** with MeSO_2_SMe provides the thiomethyl derivative **110** in 81% yield (Scheme 18 and Supporting Information File 1, Procedure 7) [46].

**Scheme 19:**
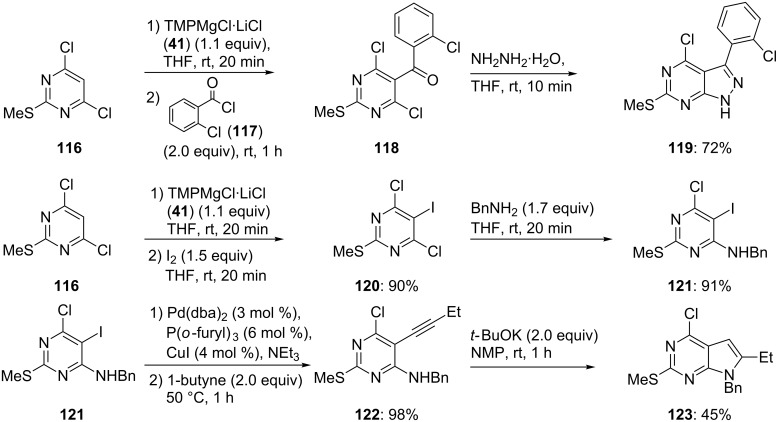
Synthesis of a p38 kinase inhibitor **119** and of a sPLA2 inhibitor **123**.

The presence of a thiomethyl substituent considerably increases the electron density of this pyrimidine and the addition of a Grignard reagent to this heterocycle can no longer occur. Therefore, a subsequent magnesiation of **110** with TMPMgCl·LiCl (1.0 equiv) can be performed at 25 °C. After 5 min reaction time at this temperature, the resulting 6-magnesiated pyrimidine **111** is obtained quantitatively. Quenching of **111** with Cl_2_FCCClF_2_ (**112**) provides the trisubstituted pyrimidine **113** in 76% yield. A final functionalization in position 5 is readily achieved by treating **113** with a further equivalent of TMPMgCl·LiCl (**41**, 25 °C, 20 min) providing the 5-magnesiated pyrimidine **114**. Quenching with benzoyl chloride furnishes the expected unsaturated ketone, which by treatment with hydrazine (NH_2_-NH_2_·H_2_O, THF, 25 °C, 10 min) leads to the pyrazolopyrimidine **115** in 56% overall yield (Scheme 18) [46]. A similar approach has been used to prepare the p38 kinase inhibitor **119** in 72% overall yield, as well as the sPLA2 inhibitor **123**, in a short reaction sequence (Scheme 19) [46].

**Scheme 20:**
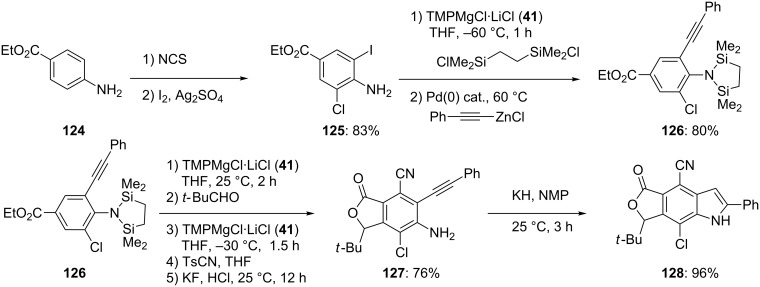
Synthesis of highly substituted indoles of type **128**.

Using TMPMgCl·LiCl (**41**), it is possible to prepare fully substituted indoles, such as **128** (Scheme 20) [47]. Thus, starting from the aniline **124**, an *ortho*-directed chlorination with *N*-chlorosuccinimide at 90 °C followed by an iodination with iodine and Ag_2_SO_4_ furnishes the tetrasubstituted aniline **125**. Protection of the free amino-group followed by a Negishi-reaction provides the scaffold **126** in 80% yield (Scheme 20).

Successive magnesiations at the positions 5 and 3 of the tetrasubstituted anilines **126** with TMPMgCl·LiCl (**41**) can be performed. The strongly electron-withdrawing properties of the chloro-substituent favor a metalation at position 5. After the addition of pivaldehyde, the subsequent addition of a second equivalent of TMPMgCl·LiCl (**41**; −30 °C, 1.5 h) allows now a magnesiation in position 3. Quenching with TsCN and deprotection of the silylated aniline with KF and HCl furnishes the hexa-substituted aniline **127** in 76% overall yield. Potassium hydride mediated ring closure in NMP [48] affords the desired indole **128** in 96% yield (Scheme 20) [47].

**Scheme 21:**
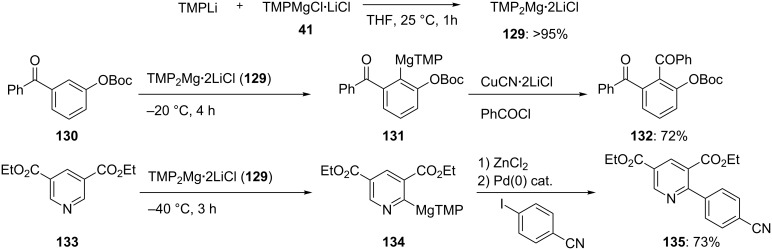
Efficient magnesiations of polyfunctional aromatics and heterocycles using TMP_2_Mg·2LiCl (**129**).

In some cases, TMPMgCl·2LiCl (**41**) is not reactive enough to achieve a magnesiation in a reasonable time frame. We therefore prepared a more reactive bis-TMP base, TMP_2_Mg·2LiCl (**129**), by mixing TMPLi with the commercially available base **41** [49]. The metalation temperature using such a base is low enough that functional groups such as a Boc-group or an aryl ketone are readily tolerated. Thus, the Boc-substituted benzophenone **130** reacts with TMP_2_Mg·2LiCl (1.1 equiv, −20 °C, 4 h) providing the expected aryl magnesium amide **131**, which after a copper-mediated benzoylation leads to the 1,2,3-trisubstituted diketone **132** in 72% yield. This reagent allows a smooth functionalization of heterocycles such as the dicarbethoxypyridine **133**, which is readily magnesiated with the base **129** at −40 °C within 3 h, leading to **134**. After a Negishi cross-coupling reaction with an aromatic iodide, the 2-functionalized pyridine **135** is obtained in 73% yield (Scheme 21, Procedure 8) [49].

### 3 New Pd- and Ni-cross-coupling procedures

Although numerous cross-coupling methods have been recently described in the literature [50–52], there is still the need for new convenient procedures. We would like to focus on the chemoselectivity problem in cross-couplings in this short section and report two protocols recently developed in our laboratories:

1. A chemoselective Negishi cross-coupling protocol tolerating acidic hydrogen atoms.
2. A chemoselective Kumada cross-coupling based on a new radical mechanism.

#### 3.1 Chemoselective Negishi cross-coupling using substrates bearing acidic hydrogen atoms

**Scheme 22:**
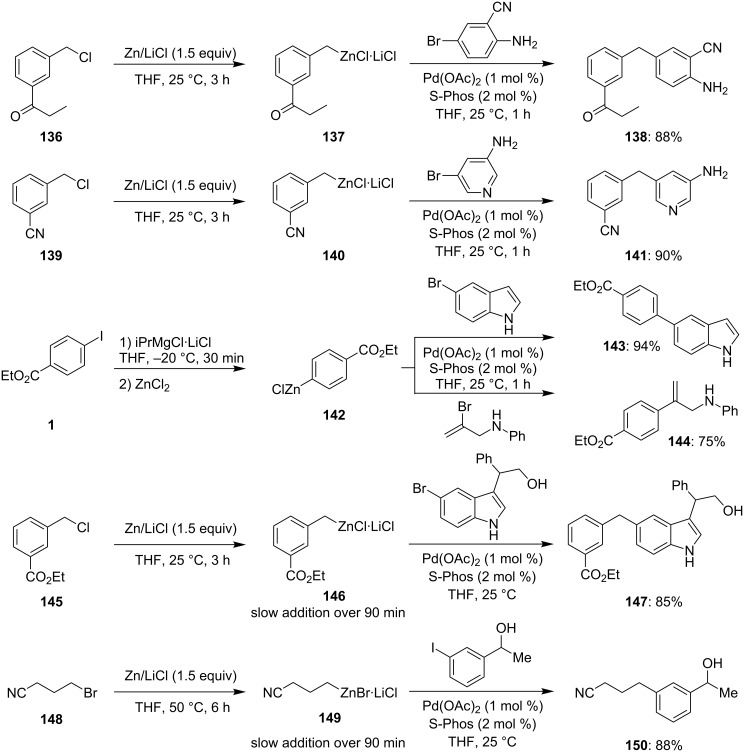
Negishi cross-coupling in the presence of substrates bearing an NH- or an OH-group.

The ability to perform cross-couplings is certainly one of the most versatile functions of heterocyclic zinc intermediates. Recently, we have shown that NiCl_2_ (0.05 mol %) constitutes an economical method for performing Negishi cross-couplings [18–19], however, it does not solve the problem of the moderate chemoselectivity of organozinc reagents towards substrates bearing acidic hydrogen atoms, such as N–H and O–H bonds. This is an important limitation of the Negishi cross-coupling, especially compared to the Suzuki cross-coupling based on boronic acid derivatives, which are much more tolerant toward acidic NH- and OH-groups. In the course of our studies, we found that by using an active catalyst system, such as S-Phos, developed by S. L. Buchwald [15–17], a smooth cross-coupling can be achieved between benzylic, aromatic and alkyl zinc reagents with substrates bearing an NH- or an OH-group (Scheme 22) [53–54].

**Scheme 23:**
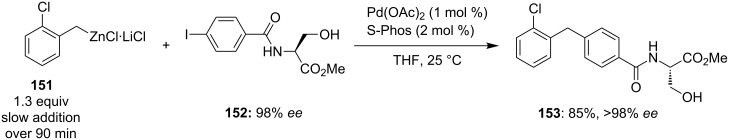
Negishi cross-coupling in the presence of a serine moiety.

Remarkably, this reaction protocol was extended to substrates bearing an α-aminoester moiety, such as **152** providing the cross-coupling product **153** in 85% yield (Scheme 23 and Supporting Information File 1, Procedure 9) [54].

#### 3.2 Radical catalyzed Kumada chemoselective cross-coupling

**Scheme 24:**
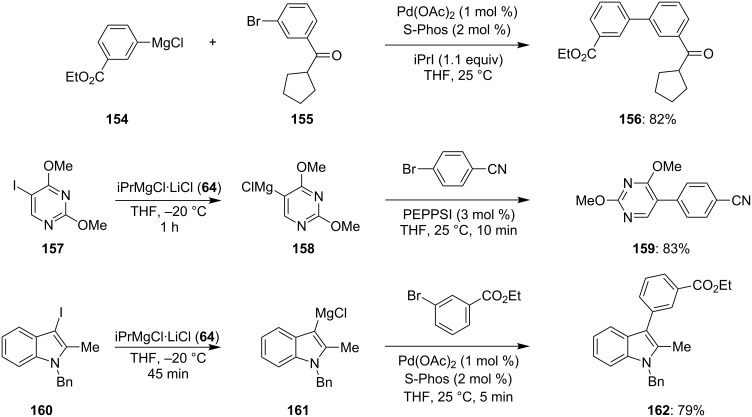
Radical catalysis for the performance of very fast Kumada reactions.

As aryl- and heteroarylmagnesium reagents are readily available, it would be highly desirable if cross-couplings could be directly realized using these organometallics without the need of further transmetalation to zinc, boron or other metals. However, the disadvantage of this cross-coupling, known in the literature as Kumada cross-coupling [55–56], is that it only proceeds with relatively nonfunctional molecules as the C–Mg bond can competitively attack the functional group present in the aromatic or heterocyclic electrophile instead of undergoing the desired cross-coupling. We have found that the presence of iPrI (or another alkyl iodide) catalyzes the Kumada cross-coupling reaction, such that highly reactive functional groups, such as ketones, esters or nitriles, are perfectly tolerated (Scheme 24 and Supporting Information File 1, Procedure 10) [57–58].

The mechanism of the reaction has been shown to be of radical nature, and it affords the cross-coupling products in very short reaction times, often less than 5 min.

### 4 MgCl_2_-Enhanced reactivity of functionalized organozincs towards their addition to aldehydes, ketones and carbon dioxide

**Scheme 25:**
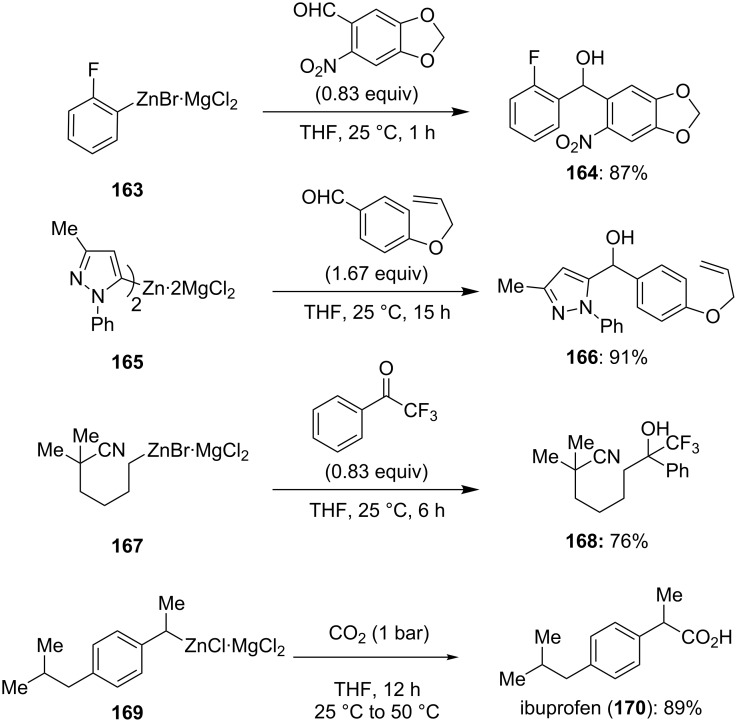
MgCl_2_-mediated addition of functionalized aromatic, heteroaromatic, alkyl and benzylic organozincs to aldehydes, ketones and carbon dioxide.

The addition reactions of organometallic reagents to ketones, aldehydes and carbon dioxide are essential transformations in organic synthesis as they provide a convenient access to various types of alcohols or carboxylic acids. Usually, organozinc reagents only react with these types of electrophiles in the presence of catalytic amounts of transition metal salts and in a very limited scope. Recently, we showed that the cheap and non-toxic main group Lewis acid MgCl_2_ allows smooth addition reactions of different aromatic, heteroaromatic, alkyl and benzylic zinc reagents to various carbonyl derivatives and carbon dioxide without the use of polar cosolvents (Scheme 25 and Supporting Information File 1, Procedure 11). The Lewis acid MgCl_2_ is usually generated during the formation of the organozinc reagent by a magnesium insertion in the presence of ZnCl_2_ (compare section 1.2) [59–60].

Thus, 2-fluorophenylzinc bromide **163** and the pyrazolylzinc chloride **165** react at room temperature with the aromatic aldehydes to provide the secondary alcohols **164** and **166** in 87–91% yield. The alkyl zinc reagent **167** adds to α,α,α-trifluoromethylacetophenone in 2 h and the corresponding alcohol **168** was isolated in 76% yield. Furthermore, the method was applied to the synthesis of the blockbuster drug ibuprofen (**170**). To achieve this, the secondary benzylic zinc reagent **169** was reacted with CO_2_ gas to provide the phenylacetic acid **170** in 89% yield.

## Conclusion

We have summarized the most important procedures for the preparation of functionalized organzinc and organomagnesium reagents in this short review. Although, these reagents were introduced to synthetic organic chemistry at the turn of the 20^th^ century, they are now more than ever essential organometallic intermediates. The progress in the 5 last years in our laboratories shows that much is still unknown in this field, and that the important synthetic preparation methods developed recently will lead to a revolution in the field and considerably expand the use of these organometallics in synthesis.

## Experimental

Experimental details for the most important reactions of this review are given in the Supporting Information File 1.

## Supporting Information

File 1 Experimental section.
