# Supplementary material for: Functionalization of heterocyclic compounds using polyfunctional magnesium and zinc reagents
Source: Beilstein J Org Chem. 2011 Sep 13;7:1261–77. doi: 10.3762/bjoc.7.147 (PMC3182436; doi:10.3762/bjoc.7.147)
Supplement: File 1 — Experimental section. [file Beilstein_J_Org_Chem-07-1261-s001.pdf]

Supporting Information

for

Functionalization of heterocyclic compounds using polyfunctional magnesium and zinc reagents

Paul Knochel, Matthias A. Schade, Sebastian Bernhardt, Georg Manolikakes, Albrecht Metzger, Fabian M. Piller, Christoph J. Rohbogner and Marc Mosrin*

Department Chemie, Ludwig-Maximilians-Universität München, Butenandtstr. 5-13, 81377 München, Germany

Email: Paul Knochel* - knoch@cup.uni-muenchen.de

* Corresponding author

Experimental section

<u>Typical procedure 1</u> for the preparation of *p*-toluenesulfonic acid 7-(2,2-dimethylpropionyl)-5-iodo(quinolin-8-yl) ester (24) [1]:

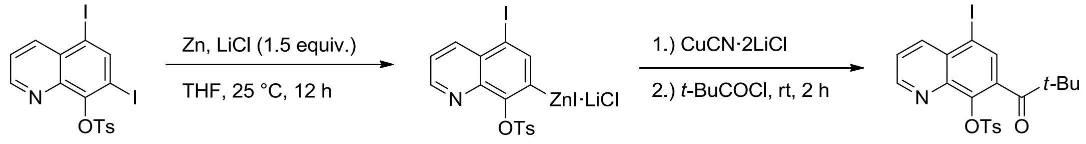

22 **23** **24**: 78%

Anhydrous LiCl (172 mg, 3 mmol) was placed in an Ar-flushed flask and dried for a further 10–20 min at 150–170 °C under high vacuum (1 mbar), or for 2–3 min at 450 °C. Zinc powder (260 mg, 3 mmol, 1.5 equiv, <10 micron, 98+%, Aldrich) was added under Ar, and the heterogeneous mixture of Zn and LiCl was dried again for 10–20 min at 150–170 °C under high vacuum (1 mbar). Afterwards the flask was evacuated and flushed with argon three times. After the addition of 1 mL of THF, Zn was activated by the treatment first with 1,2-dibromoethane (18 mg, 0.01 mL, 5 mol %) and then with chlorotrimethylsilane (4 mg, 2 mol %). *p*-Toluenesulfonic acid 5,7-diiodo(quinolin-8-yl) ester (**22**) (1.10 g, 2 mmol) was added neat at 25 °C. The reaction was stirred for 24 h at 25 °C. Then, not reacted zinc powder was allowed to settle down and the organozinc solution in THF was carefully transferred to a new, dry, Ar-flushed flask using a syringe. Then, a CuCN·2LiCl-solution (1 M in THF, 2 mL, 2 mmol) was added at 0 °C and the reaction mixture was stirred at this temperature for 10 min. Afterwards, pivaloyl chloride was added at −50 °C and the reaction was stirred for 1 h at −50 °C and then warmed to room temperature within 6 h. The reaction mixture was then quenched with sat. aqueous NH_4Cl solution. The aqueous layer was extracted with ethyl acetate. The combined organic extracts were dried with Na_2SO_4 and concentrated in vacuo. Purification by flash chromatography (pentane/dichloromethane = 1:1) afforded *p*-toluenesulfonic acid 7-(2,2-dimethylpropionyl)-5-iodo(quinolin-8-yl) ester (**24**, 78%) as a pale yellow solid.

mp 119.5–124.0 °C.

^{1}**H NMR** (400 MHz, $CDCl_3$) δ (ppm) = 8.87 (dd, ^{3}J(H,H) = 4.1 Hz, ^{3}J(H,H) = 1.5 Hz, 1H), 8.34 (dd, ^{3}J(H,H) = 8.5 Hz, ^{4}J(H,H) = 1.5 Hz, 1H), 7.90 (d, ^{3}J(H,H) = 8.3 Hz, 2H), 7.84 (s, 1H), 7.53 (dd, ^{3}J(H,H) = 8.5 Hz, ^{3}J(H,H) = 4.1 Hz, 1H), 7.43 (d, ^{3}J(H,H) = 8.4 Hz, 2H), 2.46 (s, 3H), 1.12 (s, 9H).

^{13}**C NMR** (100 MHz, $CDCl_3$) δ (ppm) = 208.4, 151.8., 145.1, 142.4, 142.3, 140.2, 136.0, 134.3, 133.8, 131.2, 129.3, 128.8, 123.9, 96.3, 45.4, 26.7, 21.7.

MS (70 eV, EI) *m/z* (%): 451.9 ((M^+−*t*-Bu); 100), 445.5 (47), 383.5 (21), 352.4 (12), 297.2 (73), 270.7 (23), 155.0 (51), 91.0 (37).

IR (KBr) $\tilde{\nu}$ (cm^{-1}): 2968 (w), 1997 (s), 1477 (w), 1451 (w),1372 (s), 1341 (m), 1206 (m), 1177 (s), 1085 (s), 999 (m), 806 (m), 779 (s), 742 (s), 688 (m), 671 (s).

HRMS (EI) for $C_{21}H_{20}INO_4S$: calcd. 509.0158; found: 509.0164

<u>Typical procedure 2</u> for the preparation of the benzylic zinc reagent 34 and its Ni-catalyzed cross-coupling with 35 leading to the pyridine derivative 36 [2]:

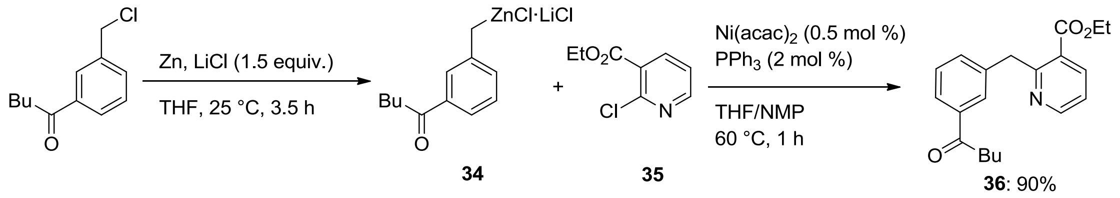

A Schlenk-flask equipped with a magnetic stirring bar and a septum was charged with LiCl (4.21 g, 30.0 mmol) and dried at 450 °C for 10 min under high vacuum. After cooling to 25 °C, the flask was evacuated and flushed with argon three times. Zinc dust (1.96 g, 30.0 mmol) was added followed by THF (5 mL). 1,2-Dibromethane was added (188 mg, 0.09 mL, 5 mol %) and the reaction mixture was heated until boiling. After cooling to 25 °C, chlorotrimethylsilane (22 mg, 0.03 mL, 1 mol %) was added and the mixture was heated

again until boiling. 3-Pentanoylbenzyl chloride (4.21 g, 20.0 mmol) was added drop wise as a solution in THF (5 mL) at 25 °C. The reaction mixture was stirred at 25 °C for 3.5 h and afterwards centrifuged for 75 min at 2000 rpm (alternatively, the reaction mixture was allowed to stand until all the zinc dust settled). Iodometric titration of the organozinc solution indicated a yield of 72%.

For the cross coupling reaction, a dry argon-flushed Schlenk flask equipped with a septum and a magnetic stirring bar, was charged with 2-chloronicotinic acid ethyl ester (**35**) (371 mg, 2.00 mmol) dissolved in NMP (0.4 mL). Subsequently, PPh_3 (0.1 mL, 0.4 M in THF, 0.40 mmol, 2 mol %) and $Ni(acac)_2$ (0.1 mL, 0.1 M in THF, 0.1 mmol, 0.5 mol %) were added. After the addition of the 3-pentanoylbenzylzinc chloride (**34**, 2.30 mL, 2.40 mmol 1.06 M in THF), the reaction mixture was warmed to 60 °C and stirred for 1 h. The reaction mixture was quenched with sat. aqueous NH_4Cl solution and extracted three times with EtOAc. The combined organic layers were washed with brine, dried over Na_2SO_4 and the solvent removed in vacuo. Purification by flash chromatography (pentane/dichloromethane = 6:1 then 1:1) afforded 2-(3-pentanoylbenzyl)nicotinic acid ethyl ester **36** (90%) as a pale yellow liquid.

^{1}H NMR (300 MHz, $CDCl_3$) δ (ppm) = 8.67 (dd, *J* = 4.9 and 1.9 Hz, 1H), 8.12 (dd, *J* = 7.9 and 1.8 Hz, 1H), 7.86 (m, 1H), 7.75 (m, 1H), 7.44 (m, 1H), 7.32 (t, *J* = 7.7 Hz, 1H), 7.24 (dd, *J* = 8.0 and 4.9 Hz, 1H), 4.63 (s, 2H), 4.32 (q, *J* = 7.1 Hz, 2H), 2.90 (t, *J* = 7.3 Hz, 2H), 1.67 (quint, *J* = 7.4 Hz, 2H), 1.37 (sext, *J* = 7.5 Hz, 2H), 1.32 (t, *J* = 7.2 Hz, 3H), 0.92 (t, *J* = 7.3 Hz, 3H).

^{13}C NMR (75 MHz, $CDCl_3$) δ (ppm) = 200.6, 166.3, 160.6, 151.9, 140.1, 138.8, 137.1, 133.6, 128.7, 128.4, 126.1, 125.9, 121.4, 61.5, 42.1, 38.3, 26.5, 22.4, 14.1, 13.9.

MS (70 eV, EI) *m/z* (%): 325 (M^+, 79), 283 (12), 282 (12), 269 (16), 268 (100), 212 (10), 211 (13), 167 (27), 166 (24).

HRMS (EI) for $C_{20}H_{23}NO_3$: calcd. 325.1678; found: 325.1666**.**

IR (ATR) $\tilde{\nu}$ (cm^{-1}): 2958 (m), 2933 (m), 2872 (w), 1719 (vs), 1681 (s), 1582 (m), 1568 (m), 1436 (m), 1366 (m), 1274 (s), 1256 (vs), 1173 (m), 1158 (m), 1130 (s), 1111 (m), 1079 (s), 1057 (m), 1018 (m), 862 (w), 776 (m), 752 (m), 741 (m), 694 (m), 629 (w), 576 (w).

Typical Procedure 3 for the preparation of ethyl 4-(2-oxo-2*H*-chromen-3-yl)benzoate (48) [3]:

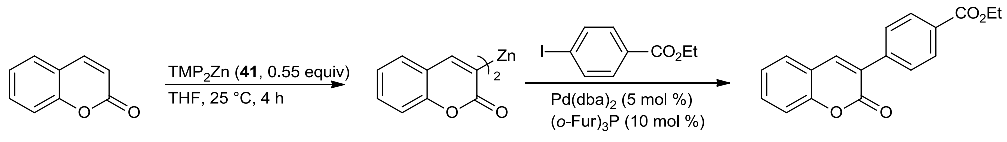

46 **47** **48**: 83%

A dry and argon flushed 10 mL Schlenk-flask, equipped with a magnetic stirring bar and a septum was charged with a solution of coumarine (**46**) (0.146 g, 1.0 mmol). The zinc base $(TMP)_2Zn{\cdot}2MgCl_2{\cdot}2LiCl$ (**41**; 0.5 M in THF, 1.10 mL, 0.55 mmol) was added dropwise at 25 °C and the reaction mixture was stirred for 4 h (0.55 mmol). The completion of the metalation was checked by GC-analysis of reaction aliquots quenched with a solution of I_2 in dry THF. $Pd(dba)_2$ (28 mg, 5 mol %) and P(*o*-furyl)$_3$ (23 mg, 10 mol %) dissolved in THF (2 mL) were then transferred through cannula to the reaction mixture, followed by the addition of ethyl 4-iodobenzoate (0.414 g, 1.5 mmol) dissolved in THF (1 mL). The reaction mixture was stirred at 25 °C for 2 h. The reaction mixture was quenched with a sat. aq. NH_4Cl solution (5 mL), extracted with diethyl ether (3 × 15 mL) and dried over anhydrous $MgSO_4$. After filtration, the solvent was evaporated in vacuo. Purification by flash-chromatography (*n*-pentane/diethyl ether, 3:1) furnished the compound **48** (0.244 g, 83%) as a colorless solid.

mp 193.3–194.4 °C.

^{1}H NMR (400 MHz, $CDCl_3$) δ (ppm) = 8.12 (d, ^{3}J = 8.5 Hz, 2H), 7.89 (s, 1H), 7.79 (d, ^{3}J = 8.7 Hz, 2H), 7.56 (m, 2H), 7.35 (m, 2H), 4.40 (d, ^{3}J = 7.2 Hz, 2H), 1.41 (t, ^{3}J = 7.0 Hz, 3H).

^{13}C NMR (100 MHz, $CDCl_3$) δ (ppm) = 166.4, 160.4, 154.0, 141.0, 139.2, 132.2, 130.9, 123.0, 128.7, 128.4, 127.6, 124.9, 119.6, 116.8, 61.4, 14.6.

MS (70 eV, EI) *m/z* (%): 295 (17), 294 (81) [M^+], 266 (23), 250 (22), 249 (100), 238 (16), 222 (11), 221 (39), 165 (45), 163 (10), 44 (26).

IR (ATR) $\tilde{\nu}$ (cm^{-1}): 1710, 1606, 1560, 1478, 1366, 1292, 1272, 1234, 1104, 954, 864, 856, 784, 766, 752, 738, 730, 698, 640, 622.

HRMS (EI) for $C_{18}H_{14}O_4$ (294.0892): 294.0915.

<u>Typical Procedure 4</u> for the preparation of ethyl 2-chloro-4-(thiophene-2-carbonyl)-nicotinate (63) [4]:

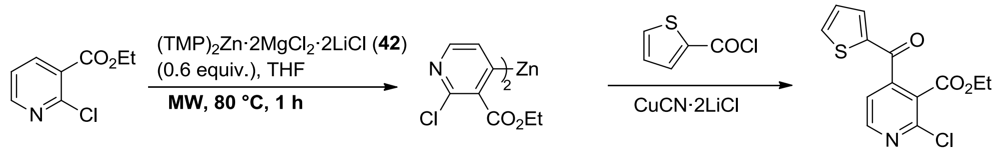

61 **62** **63**: 80%

A dry and argon flushed 10 mL pressurized vial, equipped with a magnetic stirring bar was charged with a solution of ethyl 2-chloronicotinate (**61**; 370 mg, 2.0 mmol) in dry THF (1 mL). The zinc base $(TMP)_2Zn·2MgCl_2·2LiCl$ (**42**; 0.4 M in THF, 3.00 mL, 1.2 mmol) was added and the reaction mixture was heated in a 10 mL pressurized vial, with the aid of a Discover BenchMate® Microwave system (100 W, 80 °C, 1 h). The completion of the metalation was checked by GC-analysis of reaction aliquots quenched with a solution of I_2 in dry THF. After complete metalation and cooling to room temperature, the resulting reaction mixture was put into a dry and argon flushed 25 mL Schlenk-flask, equipped with a magnetic stirring bar and

a septum. The reaction mixture was cooled to −30 °C, CuCN·2LiCl (1.0 M solution in THF, 2.2 mL, 2.2 mmol) was added and the reaction mixture was stirred for 20 min. Then, thiophene-2-carbonyl chloride (365 mg, 2.5 mmol) was added at −30 °C. The reaction mixture was slowly warmed to 25 °C and stirred at this temperature for 2 h. The reaction mixture was quenched with a sat. aq. NH_4Cl solution (10 mL), extracted with diethyl ether (5 × 15 mL) and dried over anhydrous $MgSO_4$. After filtration, the solvent was evaporated in vacuo. Purification by flash-chromatography (CH_2Cl_2) furnished the compound **63** (476 mg, 80%) as a yellow oil.

^{1}H NMR (400 MHz, $CDCl_3$) δ (ppm) = 8.56 (d, *J* = 4.9 Hz, 1H), 7.79 (dd, *J* = 4.9, 1.2 Hz, 1H), 7.44 (ddd, *J* = 3.9, 1.2, 0.4 Hz, 1H), 7.39–7.43 (m, 1H), 7.13 (ddd, *J* = 5.0, 3.8, 0.4 Hz, 1H), 4.19 (q, *J* = 7.2 Hz, 2H), 1.16 (t, *J* = 7.1 Hz, 3H).

^{13}C NMR (100 MHz, $CDCl_3$) δ (ppm) = 184.7, 164.1, 150.7, 149.5, 147.9, 141.9, 136.5, 136.0, 128.6, 127.4, 120.7, 62.6, 13.5.

MS (70 eV, EI) *m/z* (%): 295 (21) [M$^+$], 252 (26), 250 (19), 249 (33), 214 (20), 111 (100).

IR (ATR) $\tilde{\nu}$ (cm^{-1}): 2982, 1728, 1643, 1575, 1543, 1512, 1464, 1449, 1407, 1380, 1354, 1285, 1257, 1208, 1166, 1117, 1057, 1009, 925, 856, 788, 765, 725, 702, 660, 618, 599, 595, 589, 579, 564.

HRMS (EI) for $C_{13}H_{10}ClNO_3S$ (295.0070): 295.0064.

<u>Typical Procedure 5</u> for the preparation of 2-bromo-3-cyanoquinoline (67) [5]:

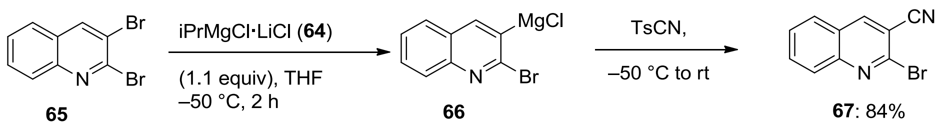

65 **66** **67**: 84%

A dry and argon flushed flask, equipped with a magnetic stirring bar and a septum, was charged with 2,3-dibromoquinoline (**65**) (1.23 g, 4.3 mmol, 1.0 equiv) dissolved in 4.3 ml dry THF. iPrMgCl·LiCl **64** (3.94 mL, 1.2 M in THF, 4.73 mmol, 1.1 equiv) was added slowly, dropwise, −50 °C. The reaction mixture was stirred at the same temperature for 2 h, the completion of the Br/Mg exchange was checked by GC-analysis, using decane as internal standard, or by TLC. Then TsCN (1.02 g, 5.6 mmol, 1.3 equiv) was added and the reaction mixture was allowed to reach 25 °C within 12 h. The consumption of the magnesium reagent was checked by GC-analysis, using decane as internal standard. After the reaction was completed, sat. NH_4Cl solution (20 mL) was added and the mixture was extracted with Et_2O (3 × 30 ml), dried over Na_2SO_4. After filtration, the solvent was removed in vacuo and the crude residue was purified by flash-chromatography (SiO_2, pentane/ether 7:3) yielding 2-bromo-3-cyanoquinoline (**67**) (837 mg, 84%) as a colorless solid.

mp 176.6–177.4 °C.

^{1}H NMR (600 MHz, $CDCl_3$) δ (ppm) = 8.50 (s, 1H), 8.10 (d, ^3J = 8.60 Hz, 1H), 7.93-7.88 (m, 2H), 7.72–7.70 (m, 1H).

^{13}C NMR (150 MHz, $CDCl_3$) δ (ppm) = 149.1, 144.5, 139.7, 134.1, 129.3, 129.0, 128.4, 125.5, 116.4, 111.1.

IR (ATR) $\tilde{\nu}$ (cm^{-1}): 3052 (s), 2230 (m), 1612 (m), 1576 (s), 1556 (s), 1486 (s), 1456 (m), 1392 (m), 1370 (s), 1358 (m), 1334 (m), 1132 (s), 1020 (vs), 1010 (s), 970 (m), 936 (s), 774 (m), 760 (vs), 678 (m).

MS (EI, 70 eV) *m/z* (%): 233 (47) [M^+, ^{81}Br], 231 (50) [M^+, ^{79}Br], 153 (10), 152 (100), 125 (21).

HRMS (EI) for ($C_{10}H_5BrN_2$) calculated 231.9636 found 231.9640.

Typical Procedure 6 for the preparation of 4'-methoxybiphenyl-2-carbonitrile (101) [6]:

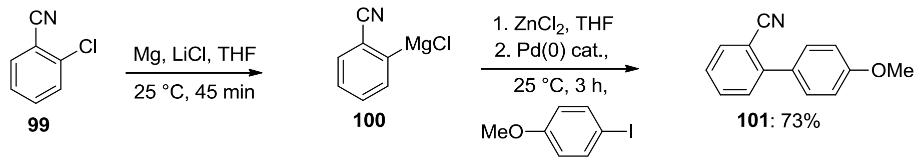

A dry and argon-flushed Schlenk-flask, equipped with a magnetic stirrer and a septum, was charged with magnesium turnings (122 mg, 5 mmol). LiCl (5.0 mL, 0.5 M in THF, 2.5 mmol) was added and the magnesium was activated with DIBAL-H (0.2 mL, 0.1 M in THF, 0.02 mmol). After 5 min of stirring 2-chlorobenzonitrile (**99**, 275 mg, 2.0 mmol) was added in one portion. The reaction mixture was stirred for 30 min and then canulated to a solution of $ZnCl_2$ (2 mL, 1 M in THF, 2.0 mmol). After 15 min $Pd(dba)_2$ (23 mg, 2 mol %), tris(*o*-furyl)phosphine (19 mg, 4 mol %) and 4-iodoanisole (328 mg, 1.4 mmol) were added and the mixture stirred for 3 h. The reaction mixture was quenched with sat. NH_4Cl solution (10 mL) and extracted with Et_2O (3 × 10 mL). The combined organic layers were dried (Na_2SO_4) and concentrated in vacuo. The crude residue was purified by flash column chromatography on silica gel (pentane/Et_2O = 20:1 to 6:1) yielding the biphenyl derivative **101** (214 mg, 73%) as a light yellow solid.

m.p.: 66 – 69 °C

^{1}H NMR (300 MHz, $CDCl_3$) δ (ppm) = 7.73 (d, J = 8 Hz, 1H), 7.61 (dt, J = 8 Hz, J = 1 Hz, 1H), 7.53–7.47 (m, 3H), 7.39 (dt, J = 8 Hz, J = 1 Hz, 1H), 7.01 (d, J = 8 Hz, 2H), 3.85 (s, 3H).

^{13}C NMR (75 MHz, $CDCl_3$) δ (ppm) = 160.1, 145.2, 133.7, 132.8, 130.5, 130.0, 129.9, 127.0, 119.0, 114.2, 111.1, 55.4.

MS (70 eV, EI) *m/z* (%): 209 (100) [M^+], 166 (45), 131 (49).

IR (ATR) $\tilde{\nu}$ (cm^{-1}): 2981, 2835, 2213, 1609, 1511, 1477, 1433, 1245, 1181, 1033, 834.

HRMS (EI) for $C_{14}H_{11}NO$ (209.0841): 209.0840.

<u>Typical Procedure 7</u> for the preparation of 2-bromo-4-(methylthio)pyrimidine (110) [7]:

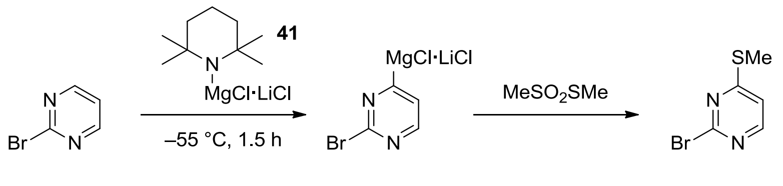

2-Bromopyrimidine (**108**) (960 mg, 6.0 mmol) dissolved in THF (6 mL) was reacted with a solution of TMPMgCl·LiCl (**41**) (1.00 M in THF, 6.6 mL, 6.6 mmol) at −55 °C for 1.5 h. *S*-Methyl methanethiolsulfonate (1.136 g, 9.0 mmol) was added dropwise at −55 °C, the resulting mixture was allowed to warm up rapidly to −30 °C and then slowly to room temperature. The reaction mixture was quenched with a sat. aq. NH_4Cl solution (50 mL), then extracted with diethyl ether (5 × 50 mL) and dried over anhydrous Na_2SO_4. After filtration, the solvent was evaporated in vacuo. Purification by flash chromatography (CH_2Cl_2/pentane 1:3) furnished the compound **110** as a white solid (981 mg, 81% yield).

mp 50.8–53.2 °C.

^{1}H NMR (300 MHz, $CDCl_3$) δ (ppm) = 8.09 (d, *J* = 5.3 Hz, 1H), 7.09 (d, *J* = 5.3 Hz, 1H), 2.54 (s, 3H).

^{13}C NMR (75 MHz, $CDCl_3$) δ (ppm) = 173.6, 155.7, 152.5, 117.4, 12.7.

MS (EI, 70 eV) *m/z* (%)**:** 206 (97), 204 (100) [^{79}Br−M$^+$], 158 (8), 124 (19), 79 (5).

IR (ATR) $\tilde{\nu}$ (cm^{-1}): 3060, 3002, 2925, 1546, 1499, 1396, 1318, 1199, 1171, 1150, 1083, 972, 830, 791, 752, 721, 672.

HRMS (EI) for $C_5H_5BrN_2S$ (203.9357): 203.9351.

Typical Procedure 8 for the preparation of *tert*-butyl 2,3-dibenzoylphenyl carbonate (132) [8]:

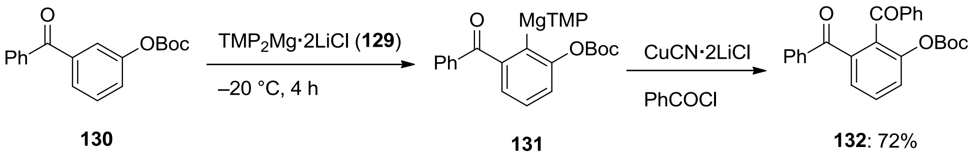

In a dry, argon flushed 10 mL Schlenk tube 3-benzoylphenyl *t*-butyl carbonate (**130**) (0.298 g, 1.0 mmol) was dissolved in 1 mL dry THF and cooled to -20 °C. Then $(TMP)_2Mg\cdot 2LiCl$ (**129**) (0.7 M in THF, 1.57 mL, 1.1 mmol) was added and the reaction mixture was stirred for 4 h. Completion of the metalation was checked by GC analysis of reaction aliquots quenched with I_2 in dry THF using decane as internal standard. Then CuCN·2LiCl (1.0 M solution in THF, 1.1 mL, 1.1 mmoL) was added at −20 °C and the reaction mixture was stirred for 15 min Thereafter, benzoyl chloride (0.264 mL, 2.2 mmol) was added at −20 °C and the reaction mixture was slowly warmed to 0 °C and stirred for 1 h. The consumption of the magnesium reagent was checked by GC-analysis, using decane as internal standard. Then sat. aq. NH_4Cl solution (10 ml) was added. The mixture was extracted with diethyl ether (3 × 25 mL) and dried over anhydrous Na_2SO_4. After filtration, the solvent was evaporated in vacuo. Purification by flash-chromatography (*n*-pentane/diethyl ether, 4:1) furnished **132** (0.305 g, 72%) as a colorless solid.

mp 138.6–140.1 °C.

^{1}H NMR (300 MHz, $CDCl_3$) δ (ppm) = 7.82–7.76 (m, 4H), 7.62–7.51 (m, 4H), 7.49–7.39 (m, 5H), 1.34 (s, 9H).

^{13}C NMR (75 MHz, $CDCl_3$) δ (ppm) = 195.6, 194.5, 151.1, 148.9, 140.0, 137.7, 134.5, 133.4, 130.5, 130.0, 129.5, 128, 6, 128.5, 128.0, 126.3, 84.3, 27.6.

MS (70 eV, EI) *m/z* (%): 303 (18), 302 (100), 301 (63) [M^+−Boc], 225 (56), 77 (34), 57 (45), 44 (13), 41 (19).

IR (ATR) $\tilde{\nu}$ (cm^{-1}): 2984, 1765, 1672, 1658, 1596, 1446, 1250, 1223, 1151, 1132, 927, 836, 700.

HRMS (EI) for $C_{25}H_{23}O_5$: calculated 403.1545 [M^++H] found 403.1154 [M^++H].

Typical procedure 9 for the preparation of ethyl 3-[3-(2-hydroxy-1-phenyl-ethyl)-1*H*-indol-5-ylmethyl]benzoate (147) [9]:

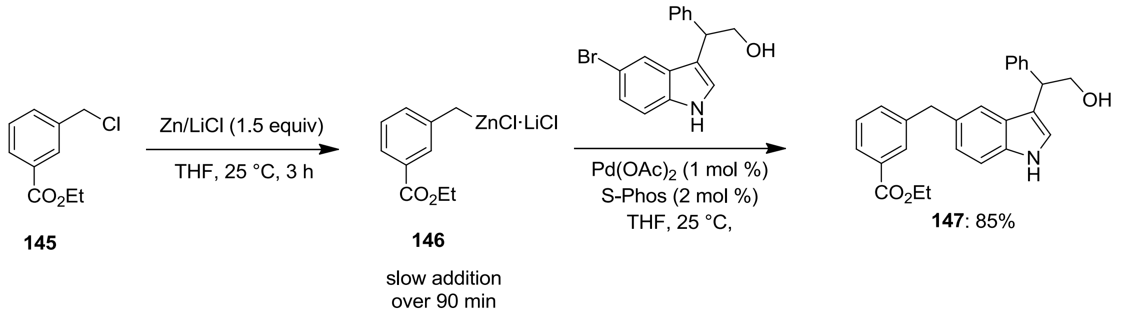

A dry and argon flushed 10 mL Schlenk-tube was charged with 2-(5-bromo-1*H*-indol-3-yl)-2-phenylethanol (632 mg, 2 mmol), $Pd(OAc)_2$ (4.5 mg, 0.02 mmol), S-Phos (16.4 mg, 0.04 mmol) and 2 mL THF. After stirring the mixture for 5 min, 3-ethoxycarbonylbenzylzinc chloride (**146**, 1.8 mL, 1.34 M in THF, 2.4 mmol) was added slowly over 90 min with a syringe pump. The reaction mixture was stirred for 1 h at 25 °C. Then the reaction mixture was quenched with a sat. NH_4Cl solution and extracted with ether. The combined organic phases were washed with an aq. solution of thiourea and dried over Na_2SO_4. Purification of the crude residue obtained after evaporation of the solvents by flash chromatography (pentane/EtOAc 1:1) yielded ethyl 3-[3-(2-hydroxy-1-phenylethyl)-1*H*-indol-5-ylmethyl]benzoate (**147**) as a colorless oil (595 mg, 85%).

^{1}H NMR (300 MHz, $CDCl_3$) δ (ppm) = 8.17 (s, 1H), 7.80–7.71 (m, 2H), 7.34–7.12 (m, 9H), 7.03 (d, *J* = 1.6 Hz, 1H), 6.96 (dd, *J* = 8.4, 1.6 Hz, 1H), 4.43 (t, J = 7.0 Hz, 1H), 4.20 (dd, *J* =

10.7, 7.0 Hz, 1H), 4.12 (dd, J = 10.7, 7.0 Hz, 1H), 4.04 (s, 2H), 2.90 (t, J = 7.4 Hz, 2H), 1.69 (m, 2H), 1.38 (m, 2H), 0.93 (t, J = 7.4 Hz, 3H).

^{13}C NMR (75 MHz, $CDCl_3$) δ (ppm) = 200.9, 142.6, 141.5, 137.1, 135.2, 133.4, 131.5, 128.5, 128.4, 128.3, 128.2, 127.2, 126.6, 125.7, 123.5, 122.3, 119.1, 115.7, 111.3, 66.3, 45.5, 41.9, 38.3, 26.5, 22.4, 13.9.

HRMS *m/z*: calc. for $C_{28}H_{29}NO_2$ 411.2198, found 411.2207.

MS (EI, 70 eV) *m/z* (%): 411 (5) [M$^+$], 396 (9), 380 (100), 294 (21), 204 (11).

IR (ATR) $\tilde{\nu}$ (cm^{-1}): 3460 (m), 3331 (m), 3027 (w), 2869 (m), 2928 (m), 2953 (m), 1666 (vs), 1601 (m), 1582 (m), 1510 (w), 1483 (m), 1452 (m), 1435 (m), 1403 (m), 1367 (m), 1274 (s), 1232 (s), 1189(m).

Typical Procedure 10 for the preparation of 4-(2,4-dimethoxypyrimidin-5-yl)benzonitrile (159) [10]:

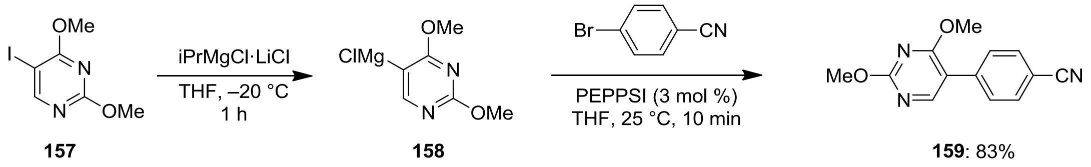

To a solution of iPrMgCl·LiCl (**64**, 3.5 mL, 3.78 mmol, 1.08 M in THF), cooled to −20 °C, was added 5-iodo-2,4-dimethoxypyrimidine (**157**, 878 mg, 3.3 mmol). The reaction mixture was stirred for 30 min at this temperature. Then, the magnesium reagent was added slowly through a teflon canula to a solution of 4-bromobenzonitrile (546 mg, 3 mmol) and PEPPSI (61.2 mg, 0.09 mmol) in 3 mL THF. The resulting mixture was stirred for 5 min at 25 °C. Then the reaction mixture was quenched with a sat. aq. solution of NH_4Cl and extracted with ether. The combined organic phases were washed with brine and dried over Na_2SO_4. Purification of the crude residue obtained after evaporation of the solvents by flash chromatography

(pentane/EtOAc 7:3) yielded 4-(2,4-dimethoxypyrimidin-5-yl)benzonitrile (**159**) as a colorless solid (601 mg, 83%).

mp 153.8–155.2 °C.

^{1}H NMR (300 MHz, $CDCl_3$) δ (ppm) = 8.25 (s, 1H), 7.66 (ddd, *J* = 8.4, 1.8, 1.7 Hz, 2H), 7.59 (ddd, *J* = 8.4, 1.8, 1.7 Hz, 2H), 4.01 (s, 3H), 4.00 (s, 3H).

^{13}C NMR (75 MHz, $CDCl_3$) δ (ppm) = 167.8, 165.4, 157.9, 138.1, 132.1, 129.2, 118.6, 114.3, 111.1, 55.0, 54.2.

HRMS *m/z*: calcd. for $C_{13}H_{11}N_3O_2$ 241.0851, found 241.0847.

MS (EI, 70 eV) *m/z* (%): 241 (100) [M^+], 226 (17), 211 (42), 169 (19), 141 (26).

IR (ATR) $\tilde{\nu}$ (cm^{-1}): 3038 (w), 2964 (w), 2361 (w), 2227 (m), 1741 (m), 1602 (s), 1563 (s), 1550 (s), 1516 (w), 1466 (s), 1400 (s), 1380 (s), 1332 (m), 1276 (m), 1234 (s), 1206 (m), 1182 (m).

<u>Typical Procedure 11</u> for the preparation of 2-fluorophenylzinc bromide (163) and (2-fluorophenyl)(6-nitro-1,3-benzodioxol-5-yl)methanol (164) [11]:

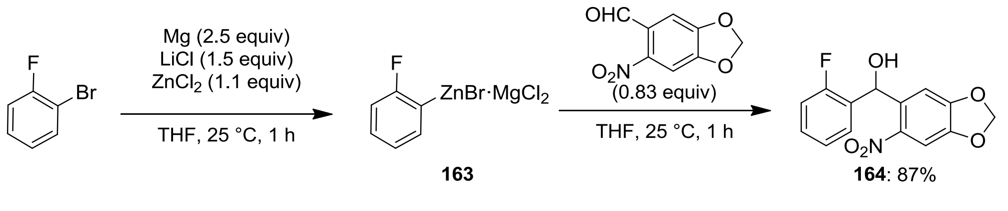

A dry and argon-flushed Schlenk-flask, equipped with a magnetic stirrer and a septum, was charged with magnesium turnings (486 mg, 20.0 mmol). Then a THF solution (10 mL) of $ZnCl_2$ (11.0 mmol) and LiCl (15.0 mmol) was added. Then a solution of 1-bromo-2-fluorobenzene (1.75 g, 10.0 mmol) in 5.0 mL THF was added dropwise using a water cooling bath to keep the temperature below 30 °C. The reaction mixture was stirred at 25 °C for 2 h

and then the supernatant solution was carefully cannulated to a new dry and argon-flushed Schlenk-flask through a syringe filter. Iodometric titration of the zinc reagent **163** indicated a concentration of 0.52 M.

A dry and argon-flushed Schlenk-flask, equipped with a magnetic stirrer and a septum, was charged with a solution of 6-nitro-1,3-benzodioxole-5-carbaldehyde (293 mg, 1.50 mmol) in 2 mL THF. Then, 2-fluorophenylzinc bromide·2$MgCl_2$ (**163**; 3.30 mL, 1.80 mmol, 0.52 M in THF) was added dropwise. The reaction mixture was stirred for 1 h at 22 °C. Then, the reaction mixture was cooled to 0 °C and quenched with sat. aq. NH_4Cl solution and extracted with EtOAc (3 x 50 mL). The combined organic phases were dried ($MgSO_4$). Evaporation of the solvents in vacuo and purification by column chromatography (silica gel, pentane / Et_2O = 7:3) afforded the alcohol **164** (378 mg, 87%) as a yellow oil.

^{1}H NMR (300 MHz, $CDCl_3$) δ (ppm) = 7.50 (s, 1H), 7.38–7.31 (m, 1H), 7.30–7.24 (m, 1H), 7.12 (td, *J* = 7.6, 1.2 Hz, 1H), 7.05–6.99 (m, 2H), 6.62 (s, 1H), 6.11–6.09 (m, 2H), 3.11 (br s, 1H).

^{13}C NMR (75 MHz, $CDCl_3$) δ (ppm) = 159.9 (d, *J* = 247.5 Hz), 152.2, 147.3, 142.1, 135.1, 129.7 (d, *J* = 8.3 Hz), 128.7 (d *J* = 20.7 Hz), 127.7 (d, *J* = 3.9 Hz), 124.2 (d, *J* = 7.6 Hz), 115.4 (d, *J* = 21.4 Hz), 108.2, 105.6, 103.7, 65.7 (d, *J* = 3.9 Hz).

IR (ATR) $\tilde{\nu}$ (cm^{-1}): 3387 (br), 3071 (w), 2990 (w), 2915 (w), 1737 (w), 1616 (w), 1586 (w), 1519 (s), 1504 (s), 1481 (vs), 1455 (s), 1422 (m), 1330 (s), 1256 (vs), 1173 (m), 1120 (m), 1095 (m), 1028 (vs).

MS (EI, 70 eV) *m/z* (%): 291 (M^+, 8), 273 (25), 228 (31), 171 (100), 157 (28).

HRMS (EI) for $C_{14}H_{10}FNO_5$: calc.: 291.0543; found: 291.0567 (M^+).

References:

1. Boudet, N.; Sase, S.; Sinha, P.; Liu, C.-Y.; Knochel, P. *J. Am. Chem. Soc.* **2007**, *129*, 12358–12359.

2. Schade, M .A.; Metzger, A.; Hug, S.; Knochel, P. *Chem. Commun.* **2008**, 3046–3048.

3. Wunderlich, S. H.; Knochel, P. *Angew. Chem., Int. Ed.* **2007**, *46*, 7685–7688.

4. Wunderlich, S. H.; Knochel, P. *Org. Lett.* **2008**, *8*, 4705–4707.

5. Boudet, N.; Lachs, J. R.; Knochel, P. *Org. Lett.* **2007**, *9*, 5525–5528.

6. Piller, F. M.; Appukkuttan, P.; Gavryushin, A.; Helm, M.; Knochel, P. *Angew. Chem., Int. Ed.* **2008**, *47*, 6802–6806.

7. Mosrin, M.; Knochel, P. *Org. Lett*. **2008**, *10*, 2497–2500.

8. Clososki, G. C.; Rohbogner, C. J.; Knochel, P. *Angew. Chem., Int. Ed.* **2007**, *46*, 7681–7684.

9. Manolikakes, G.; Schade, M. A.; Munoz Hernandez, C.; Mayr, H.; Knochel, P. *Org. Lett.* **2008**, *10*, 2765–2768.

10. Manolikakes, G.; Knochel, P. *Angew. Chem. Int. Ed.* **2009**, *48*, 205–209.

11. Metzger, A.; Bernhardt, S.; Manolikakes, G.; Knochel, P. *Angew. Chem., Int. Ed.* **2010**, *49*, 4665–4668.
